# Prospective intraoperative and histologic evaluation of cavernous sinus medial wall invasion by pituitary adenomas and its implications for acromegaly remission outcomes

**DOI:** 10.1038/s41598-022-12980-1

**Published:** 2022-06-15

**Authors:** Ahmed Mohyeldin, Laurence J. Katznelson, Andrew R. Hoffman, Karam Asmaro, Saman S. Ahmadian, Mostafa M. Eltobgy, Jayakar V. Nayak, Zara M. Patel, Peter H. Hwang, Juan C. Fernandez-Miranda

**Affiliations:** 1 Department of Neurosurgery, Stanford University Medical Center, Stanford, California USA; 2 Department of Neurosurgery, University of California-Irvine, Orange, California USA; 3 Department of Medicine, Stanford University Medical Center, Stanford, California USA; 4 Department of Pathology, Stanford University Medical Center, Stanford, California USA; 5 Department of Microbiology, Ohio State University, Columbus, Ohio USA; 6 Department of Otolaryngology, Stanford University Medical Center, Stanford, California USA

**Keywords:** Tumour heterogeneity, Pituitary tumours

## Abstract

Recurrence and biochemical remission rates vary widely among different histological subtypes of pituitary adenoma. In this prospective study, we evaluated 107 consecutive primary pituitary adenomas operated on by a single neurosurgeon including 28 corticotroph, 27 gonadotroph, 24 somatotroph, 17 lactotroph, 5 null-cell and 6 plurihormonal. In each case, we performed direct endoscopic intraoperative inspection of the medial wall of the cavernous sinus, which was surgically removed when invasion was visualized. This was performed irrespective of tumor functional status. Medial wall resection was performed in 47% of pituitary adenomas, and 39/50 walls confirmed pathologic evidence of invasion, rendering a positive predictive value of intraoperative evaluation of medial wall invasion of 78%. We show for the first-time dramatic disparities in the frequency of medial wall invasion among pathological subtypes. Somatotroph tumors invaded the medial wall much more often than other adenoma subtypes, 81% intraoperatively and 69% histologically, followed by plurihormonal tumors (40%) and gonadotroph cell tumors (33%), both with intraoperative positive predictive value of 100%. The least likely to invade were corticotroph adenomas, at a rate of 32% intraoperatively and 21% histologically, and null-cell adenomas at 0%. Removal of the cavernous sinus medial wall was not associated with permanent cranial nerve morbidity nor carotid artery injury, although 4 patients (all Knosp 3-4) experienced transient diplopia. Medial wall resection in acromegaly resulted in the highest potential for biochemical remission ever reported, with an average postoperative day 1 GH levels of 0.96 ug/L and surgical remission rates of 92% based on normalization of IGF-1 levels after surgery (mean = 15.56 months; range 3–30 months). Our findings suggest that tumor invasion of the medial wall of the cavernous sinus may explain the relatively low biochemical remission rates currently seen for acromegaly and illustrate the relevance of advanced intradural surgical approaches for successful and durable outcomes in endonasal pituitary surgery for functional adenomas.

## Introduction

The recent reclassification of pituitary tumors by the World Health Organization (WHO) in 2017 organized adenomas according to their cell lineage to better account for their heterogenous clinical and endocrinological presentations^1–3^. As distinct patterns of disease are determined under this paradigm, a persistent challenge in pituitary surgery exists. Surgical outcomes and recurrence are profoundly influenced by invasion into parasellar tissues, particularly the cavernous sinus^4–6^, yet fundamental insights into these mechanisms are incompletely understood^5,7–10^.

The pituitary gland is separated from the venous sinus spaces by the medial wall of the cavernous sinus, a contiguous single layer of meningeal dura^11–13^. The extent of parasellar invasion through the medial wall and into the cavernous sinus by pituitary adenomas remains a significant predictor of incomplete surgical resection rates, failed biochemical remission and tumor recurrence^4–6,9,14,15^. Preoperative radiographic grading scales that utilize magnetic resonance imaging, such as the Knosp classification, estimate the probability of invasion into the cavernous sinus with increasing grade^5,16^. Despite continuous revision of this classification^6^, distinguishing cavernous sinus compression from invasion remains an intraoperative assessment and as a result has led to a wide range of reported invasion frequencies among adenomas^4,5,7,9,17,18^. With the integration of high-resolution and angled endoscopes in transsphenoidal surgery, there is now strong evidence that identifying cavernous sinus invasion is best evaluated via direct visualization of the medial wall of the cavernous sinus with endoscopes over the use of intraoperative microscopes^5,16^.

Deliberate resection of the medial wall of the cavernous sinus has long been considered morbid and unattainable, which has contributed to its controversy in pituitary surgery^12,13^. Oldfield first recognized the medial wall of the cavernous sinus as a frequent nidus for recurrence in Cushing’s disease and advocated for its removal^4,19,20^. Recent laboratory investigations have better elucidated the microsurgical anatomy of the medial wall of the cavernous sinus. By defining the parasellar ligaments that tether the wall to cavernous sinus structures, this allowed for the development of an innovative surgical technique for its safe removal^12,13^. The use of histological analysis of the medial wall itself has further advanced the visualization of microscopic invasion^13,21,22^, a more precise way of assessing cavernous sinus invasion compared to sellar floor dural sampling^4,5,8,20,23^ or visualization of clival recess invasion as previously performed by other groups^8,24^. Medial wall histological analysis allows for improved validation and understanding of the limitations of current preoperative radiographic classification systems that suggest invasion while identifying adenoma subtypes with a predilection for cavernous sinus invasion. Until now, a systematic and quantified approach using this strategy has not been published.

In this study, using direct intraoperative visualization of the medial wall followed by histopathological analysis for microscopic tumor invasion, we discover that the different pituitary adenoma subtypes have varying predilections to invade the cavernous sinus. We also demonstrate that surgical resection of the medial wall is safe and may offer the best chance at successful treatment in acromegaly with 92% biochemical remission rate with surgery alone. Finally, this data draws into question whether current preoperative imaging grading systems^5,16^ can be broadly applied to all histological subtypes of pituitary adenomas in predicting cavernous sinus invasion.

## Materials and methods

### Study design and patient selection

A single-center, observational, prospective cohort study was conducted to examine differential rates of invasion of the medial wall of the cavernous sinus in patients with newly diagnosed pituitary adenomas (n=107) who underwent surgery at our institution from July 2018 to December 2020. All patients signed informed consent for evaluation and treatment. This study was performed under a protocol (#12625) approved by the institutional review board at Stanford University. All methods and experiments were performed in accordance with the relevant guidelines and regulations. In all patients, the diagnosis of pituitary adenoma was confirmed histologically. Patients with recurrent pituitary adenomas were excluded from this cohort due to the increased incidence of cavernous sinus invasion in this patient population. All patients underwent neurological and endocrinological evaluations before and after surgery. Follow-up for the acromegaly cohort ranged from 3 months to 30 months (mean = 15.56 months).

### Magnetic resonance imaging and knosp classification assessment

All patients underwent high resolution Magnetic Resonance Imaging (MRI) with and without gadolinium using conventional T1 and T2 spin echo sequences using a 3-tesla scanner. All preoperative MRI scans were reviewed and Knosp grading was performed independently by authors AM and JFM based on published Knosp criteria^5,16,25^. Discrepancies in grading between the reviewers for any given scan were revaluated by AM and JFM until a consensus grade was reached. Maximal tumor diameter was measured on preoperative imaging studies and taken as the largest measurement in the coronal, axial, or sagittal planes.

### Endocrine investigations

Biochemical remission was defined based on 2010 Acromegaly consensus criteria^26^, and all patients were evaluated and supervised by neuro-endocrinologists (LK and AH). Biochemical remission was defined when Insulin-like Growth Factor (IGF)-1 levels were normal and either their Growth Hormone (GH) nadir was less than 0.4 ng/mL during an Oral Glucose Tolerance Test (OGTT) or their random GH was less than 1.0 ng/mL on last follow-up. In circumstances where there was discordance between the patient’s OGTT (normal) and IGF-1 level (abnormal) at first follow-up, the IGF-1 level was drawn again at 6 months because of previously reported observations that IGF-1 levels can continue to decrease over time. In these cases, the patient was considered in remission if IGF-1 levels were normal at 6-12 months. In cases where there was observed discordance between the IGF-1 and OGTT beyond 12 months, the patient was deemed to have failed to attain remission.

### Surgical technique and intraoperative evaluation of cavernous sinus invasion

**Figure 1:**
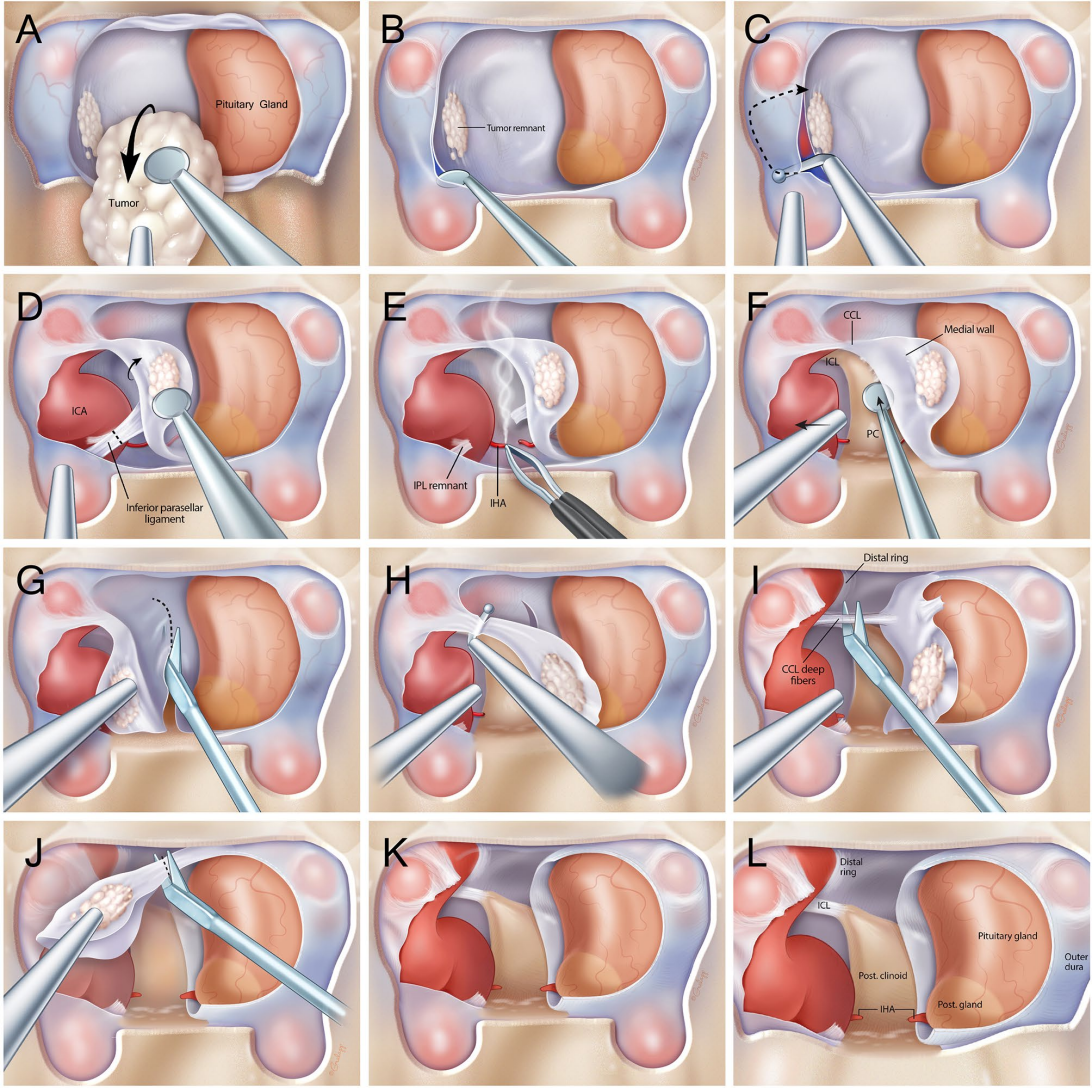
Medical illustrations demonstrating the sequential steps of resecting the medial wall of the cavernous sinus. Wide exposures of the anterior cavernous sinus and paraclinoid carotid artery allow for adequate visualization and safe removal of the medial wall. (**A**) Pituitary adenoma is resected under high resolution endoscopy with bimanual technique preserving the normal pituitary gland (**B**) The medial wall is adequately visualized with high resolution angled endoscopes once the bulk of the adenoma has been removed, tumor remnants that are intraoperatively viewed to adhere strongly to the medial wall suggests invasion necessitating resection. The anterior cavernous sinus is entered by developing a plane between the 2 layers of dura of the intercavernous sinus (**C**) Using a right angled blunt tipped feather blade, the anterior cavernous sinus wall is cut and hemostasis is achieved (**D**) The inferior parasellar ligament (IPL) is one of the first ligaments encountered and is cut to allow mobilization of the medial wall away from the carotid artery (**E**) The inferior hypophyseal artery is often encountered next and is coagulated and cut to avoid any evulsions off the carotid artery (**F**) the dura overlying the base of the posterior clinoid forms the posterior wall of the cavernous sinus and is dissected off the clinoid till the surgeon encounters the horizontal fibers of the carticoclinoidal ligament (CCL) (**G**) Microscissors are used to cut the dura covering the dorsum sella to deatch the meial wall from its sellar attachments (**H**) Using the right angled feather blade, the CCL fibers are cut to begin detaching the medial wall from the carotid artery (**I**) There are often deep fibers of the CCL that require further transection to completely untether the medial wall from the carotid artery (**J**) Once all the fibers of the CCL are cut, the medial wall is now completely free from any attachments to the carotid artery and the only remaining cut are dural attachments that make up the proximal dural ring (**K**) The medial wall is now completely free from attachments and often sent *en bloc* if it is not severely distorted by tumor invasion leaving behind and open cavernous sinus with an exposed carotid artery. (**L**) The remaining view should show the medial surface of the carotid artery in the cavernous sinus with visualization of the superior compartment of the cavernous sinus and the interclinoidal ligament (ICL).

The standard endoscopic endonasal approach to the sphenoid sinus was performed as previously described^12,13^. The step-by-step surgical technique is depicted in Fig. 1A–L. Extensive sellar and parasellar exposure, including the anterior wall of the cavernous sinus and clinoidal segment of the ICA on the side(s) of interest was completed. Once the pituitary adenoma had been adequately removed from the sella, the medial wall was inspected via high-resolution endoscopy. Medial wall invasion was preoperatively evaluated using Knosp criteria by AM and JFM, and a decision was ultimately rendered by the senior neurosurgeon (JFM) intraoperatively. Tumor elements adherent to the medial wall suggested invasion, necessitating medial wall resection. Destruction/erosion of portions of the medial wall also suggested invasion into the cavernous sinus, and prompted medial wall resection and exploration of the cavernous sinus for complete tumor removal whenever possible. If the medial wall was observed to be smooth without perforations, trabeculae or gross tumor seeding, then this was deemed to be a medial wall without invasion.

An intraoperative doppler was used to map the carotid artery prior to cavernous sinus entry and intradural cavernous sinus dissection. The cavernous sinus was typically entered at its most anterior and inferior point by developing a plane between the 2 divergent layers of dura with blunt dissection. The inner or meningeal dural layer covers the gland and forms the medial wall of cavernous sinus, while the outer or periosteal layer covers the meningeal layer anteriorly but diverges laterally to form the anterior wall of the cavernous sinus. Using a right angled blunt-tipped feather blade, the anterior cavernous sinus wall was cut laterally and superiorly to gain wide access. Venous hemostasis was readily achieved with hemostatic matrix (Floseal™, Baxter Inc) and gentle cottonoid packing before proceeding with resection. The inferior parasellar ligament (IPL) was typically the first ligament encountered and its transection allowed mobilization of the medial wall. The inferior hypophyseal artery was often encountered just behind the IPL and was coagulated and transected to avoid risk of avulsion off the ICA and facilitate further mobilization of the medial wall away from the ICA. Occasionally, tight adhesions between the ICA and tumor embedded within the medial wall made this lateral disconnection technically challenging. Great care was taken at this stage, often requiring a piecemeal resection with bipolar coagulation of remnants attached to the ICA. The medial wall was transected posteriorly at the base of the posterior clinoid where it continues as the posterior wall of the sella. Next, the medial wall was separated superiorly using a right-angled feather blade to transect the fibers of the carotidoclinoidal ligament (CCL). Transection of deep fibers of the CCL was required to completely untether the medial wall from the ICA, which. was then submitted for histological analysis. Based on retrospective review of recorded surgical videos, the documented time for exposure and resection of the medial wall of the cavernous sinus is variable depending on the degree of cavernous sinus involvement by the tumor. For low Knosp grade tumors (Knosp 0-2), we estimate the operative time of this additional step to be about 30-45 minutes extra to the standard operative time. For high Knosp grade tumors (Knosp 3-4), we estimate the operative time of this additional step to be about 60-90 minutes extra to the standard operative time.

### Histological and immunostaining assessment of the medial wall of the cavernous sinus

The resected medial wall of the cavernous sinus was collected independently from the primary pituitary tumor, fixed in formalin and sent for histological examination and evaluated independently by a neuropathologist. This dural tissue was inspected for invading pituitary tumors cells under a microscope, and immunohistochemistry was performed for transcription factors and cell markers based on the WHO 2017 criteria for each pituitary adenoma subtype. Given the conflicting reports in the literature regarding Ki-67 index labeling and pituitary adenoma phenotypes, we chose variables recognized by the WHO 2017 as distinguishing hallmarks of pituitary adenoma subtypes by focusing our resources on transcription factor profiling over Ki-67 indexing^3,27–30^.

### Statistical analysis

Logistic regression was performed using SPSS to investigate the effects of age, gender, tumor diameter, clinical syndrome/functionality, Knosp grade and pituitary adenoma subtype (as independent factors) on the likelihood that pituitary tumor invades the medial wall of cavernous sinus (a dependent variable). The logistic regression model was statistically significant when p < 0.05.

## Results

### Patient cohort and tumor characteristics

Of 107 consecutive patients with newly diagnosed pituitary adenoma treated with endoscopic transsphenoidal surgery, 62 (58%) were female (Table 1). The mean age for this cohort was 49 ± 15 years. The average tumor size was 20 ± 10.3mm.

**Table 1:** Patient Cohort n=107.

Age (years)	Mean	SD
	49.04	15.26
Tumor size (mm)	Mean	SD
	19.95	10.33
Gender	N	percent
F	62	58%
M	45	42%
Intraoperative medial wall invasion	N	Percent
Negative	57	53%
Positive	50	47%
Histological medial wall invasion	N	Percent
Negative	11	10.3%
Positive	39	36.4%
"N/A" = Negative	57	53.3%
Clinical.Syndrome	N	Percent
Functional	55	51%
Non-functional	52	49%
WHO 2017 Adenoma Classification	N	Percent
Corticotroph	28	26%
Gonadotroph	27	25%
Somatotroph	24	22%
Lactotroph	17	16%
Null cell	5	5%
Plurihormonal	6	9%
Knosp grade	N	Percent
Grade 0	28	26%
Grade 1	43	40%
Grade 2	18	17%
Grade 3A	11	10%
Grade 3B	5	5%
Grade 4	2	2%

Cohort of 107 consecutive primary pituitary adenoma patients. Variables include age, tumor size, gender, intraoperative evaluation of medial wall invasion, histological evaluation of medial wall invasion, WHO 2017 Adenoma classification and Knosp grade. Categorical variables are reported as number and percentage, and continuous variables are reported as mean ± standard deviation.

### Radiological and endocrinological features

In this cohort, 55 patients had endocrinologically functional tumors (51%) and 52 patients had non-functional tumors (49%). Based on the 2017 WHO classification, corticotroph adenomas had the largest representation in this cohort with 28 patients (26%) followed by gonadotroph adenomas (27 patients,25%), somatotroph adenomas (24 patients, 22%), lactotroph adenomas (17 patients, 16%), plurihormonal adenomas (6 patients, 6%) and null cell adenomas (5 patients, 5%). Among 107 patients, 18 (17%) fulfilled the criteria for Knosp grade 3 or 4, while 86 (83%) fulfilled the criteria for Grade 0-2. Grade 1 Knosp tumors had the most common representation in the cohort with 43 patients (40%).

### Intraoperative endoscopic evaluation of invasion of the medial wall of the cavernous sinus

In each case, we performed direct intraoperative inspection of the medial wall of the cavernous sinus, which as noted previously was surgically removed when invasion was suspected via direct intraoperative endonasal examination. Tissue resection was performed irrespective of the functional status of the tumor, patient’s age or any other parameters. Medial wall resection was performed in 50 out of 107 (47%) consecutive primary pituitary adenomas.

Binary logistic regression analyses identified three independent variables that reached statistical significance for intraoperative suspected medial wall invasion: Knosp grade, gender, and pituitary adenoma subtype (Table 2). Tumors with a Knosp grade >2 predicted invasion into the medial wall of the cavernous sinus (OR 31.64, CI 5.31-188.56, *p*=0.002), male gender had a significant positive correlation (OR 3.62, CI 1.17-11.15, *p*=0.03), and somatotroph adenomas were much more likely to be observed invading the medial wall (OR 7.89, CI 1.36-45.8, *p*=0.02). Of note, tumor size (OR 1.04, CI 0.97-1.10, *p*=0.26) and endocrine functional status (OR 4.9, CI .69-34.76, *p*=0.11) did not reach statistical significance in our analysis (Table 2). In our cases series, we only had 1 patient who did not have intraoperative cavernous sinus invasion despite extensive gland exploration and did not achieve biochemical remission; this was a case of MRI negative Cushing’s Disease.

**Table 2:** Intraoperative medial wall invasion.

Variable	Category	Odds ratio	lower CI	upper CI	*P* value
Age (years)		1.02	0.98	1.06	0.42
Gender	F	–	–	–	–
	M	3.62	1.17	11.15	**0.03**
Tumor size (mm)		1.04	0.97	1.10	0.26
Clinical Syndrome	Non-functional	–	–	–	–
	Functional	4.9	0.69	34.76	0.11
WHO 2017 adenoma classification	Corticotroph	–	–	–	–
	Gonadotroph	1.08	0.22	5.27	0.93
	Somatotroph	7.89	1.36	45.8	**0.02**
	Lactotroph	1.25	0.20	7.97	0.82
	Other	1.92	0.32	11.55	0.47
Knosp Grade	<=2	–	–	–	–
	>2	31.64	5.31	188.56	**0.0002**

Multivariate analysis of intraoperative evaluation of invasion of the medial wall of the cavernous sinus to identify variables that influence invasion. Odds ratio, Confidence Intervals and p values area documented.

### Histopathology and examination of the cavernous sinus medial wall

Of the 50 examined medial wall specimens, 39 were found to contain invading tumor cells and were designated “positive” on histological evaluation, rendering a positive predictive value of 78% for the intraoperative endoscopic evaluation of medial wall invasion. Medial walls that did not demonstrate invading tumor cells (6/11), or were confounded by coagulation artifact (5/11) and did not stain positively for tumor cells were designated “negative” (n=11). Cavernous sinus medial walls that were deemed negative upon intraoperative assessment (n=57) and left unresected were designated “N/A.” These patients were combined with the histologically “negative” group of 11 samples above, thus providing 68 patients for statistical analysis.

**Figure 2:**
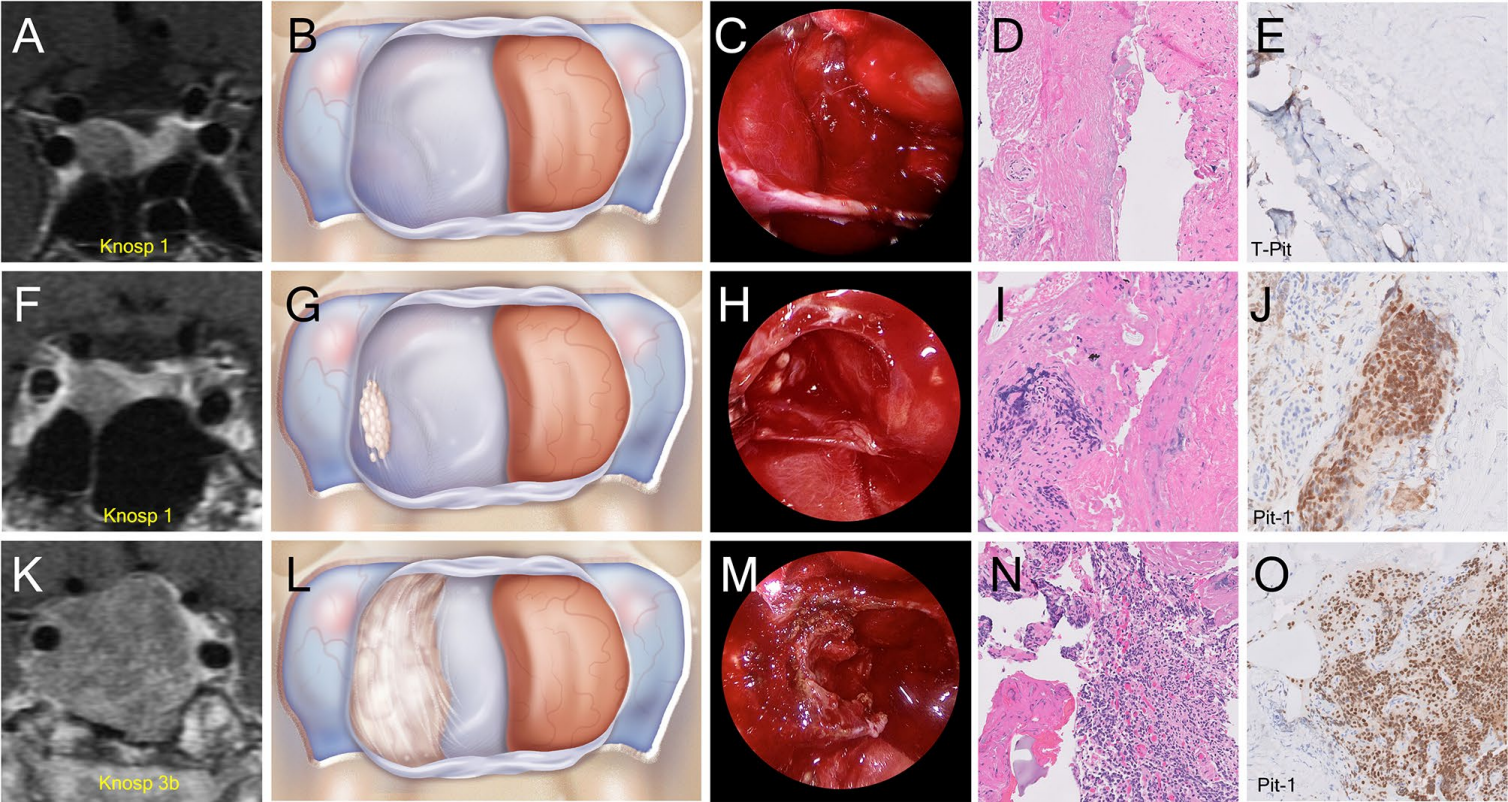
We hypothesized from our intraoperative experience with pituitary tumors that some pituitary adenoma subtypes have a predilection for invading the medial wall of the cavernous sinus more so than others. Case examples of various pituitary adenomas with varying Knosp grade and invasion into the medial wall of the cavernous as viewed on preoperative MRI (T1 with gadolinium), through medical illustrations, from an endoscopic intraoperative view, and microscopically with histological H&E staining and immunocytochemistry. (**A**) Preoperative coronal MRI demonstrating a Knosp grade 1 corticotroph adenoma (**B**) A medical illustration of the intraoperative view from an endoscopic transsphenoidal approach after the adenoma has been removed and the pituitary gland remains displaced the left side of the field exposing an intact medial of the cavernous sinus with no evidence of invasion (**C**) An intraoperative view from an endoscopic transsphenoidal approach after the adenoma has been removed and the pituitary gland remains displaced the left side of the field exposing an intact right medial wall of the cavernous sinus with no evidence of invasion (**D**) Histological images of H&E slides of a resected medial wall with no evidence of invading pituitary adenoma cells (**E**) Histological images of immunohistochemistry slides of a resected medial wall with no evidence of invading pituitary adenoma cells (staining for T-pit transcription factor) (**F**) Preoperative coronal MRI demonstrating a Knosp grade 1 somatotroph adenoma (**G**) A medical illustration of the intraoperative view from an endoscopic transsphenoidal approach after the adenoma has been removed and the pituitary gland remains displaced to the left side of the field exposing the right medial wall of the cavernous sinus with subtle evidence of tumor invasion (**H**) An intraoperative view from an endoscopic transsphenoidal approach after the adenoma has been removed and the pituitary gland remains displaced the left side of the field exposing the right medial wall of the cavernous sinus with subtle evidence of tumor invasion (**I**) Histological images of H&E slides of the resected medial wall with evidence of invading pituitary adenoma cells (**J**) Histological images of immunohistochemistry slides of the resected medial wall in the case with evidence of invading pituitary adenoma cells (staining for Pit-1 transcription factor) (**K**) Preoperative coronal MRI demonstrating a Knosp grade 3 somatotroph adenoma (**L**) A medical illustration of the intraoperative view from an endoscopic transsphenoidal approach after the adenoma has been removed and the pituitary gland remains displaced to the left side of the field exposing the right medial wall of the cavernous sinus with frank evidence of tumor invasion (**M**) An intraoperative view from an endoscopic transsphenoidal approach after the adenoma has been removed and the pituitary gland remains displaced the left side of the field exposing the right medial wall of the cavernous sinus with frank evidence of tumor invasion (**N**) Histological images of H&E slides of the resected medial wall in this case with evidence of invading pituitary adenoma cells O. Histological images of immunohistochemistry slides of the resected medial wall in this case with evidence of invading pituitary adenoma cells (staining for Pit-1 transcription factor).

Binary logistic regression analyses identified two variables that reached statistical significance in predicting histological invasion: Knosp grade and pituitary adenoma subtype (Table 3). Tumors with a Knosp grade >2 (OR 50.04, CI 29.01-111.23, *p*=0.0002) and somatotroph adenomas (OR 20.6, CI 2.55-167.88, *p*<0.05) were highly predictive of histological invasion. Neither gender (OR 2.46, CI 0.83-7.3, *p*=0.11), tumor size (OR 1.00, CI 0.95-1.07, *p*=0.69) nor functional status of the tumor (OR 1.27, CI 0.14-12.00, *p*=0.83) achieved statistical significance in our analysis and therefore did not predict cavernous sinus medial wall invasion (Table 3). Figure 2 demonstrates case examples of different pituitary adenomas with varying preoperative Knosp grades as well as the same Knosp grade demonstrating differential patterns of invasion into the medial wall of the cavernous sinus.

**Table 3:** Histological Medial Wall Invasion.

Variable	Category	Odds ratio	lower CI	upper CI	*P* value
Age (years)		1.02	0.98	1.06	0.45
Gender	F	–	–	–	–
	M	2.46	0.83	7.30	0.11
Tumor size (mm)		1.00	0.95	1.07	0.69
Clinical Syndrome	Non-functional	–	–	–	–
	Functional	1.27	0.14	12.00	0.83
WHO 2017 adenoma classification	Corticotroph	–	–	–	–
	Gonadotroph	2.26	0.41	12.5	0.35
	Somatotroph	20.66	2.55	167.68	0.05
	Lactotroph	5.76	0.60	54.97	0.13
	Other	1.70	0.20	14.82	0.63
Knosp grade	<=2	–	–	–	–
	>2	50.04	29.01	111.23	0.0002

Multivariate analysis of histological evaluation of invasion of the medial wall of the cavernous sinus to identify variables that influence invasion. Odds ratio, Confidence Intervals and p values area documented.

**Figure 3:**
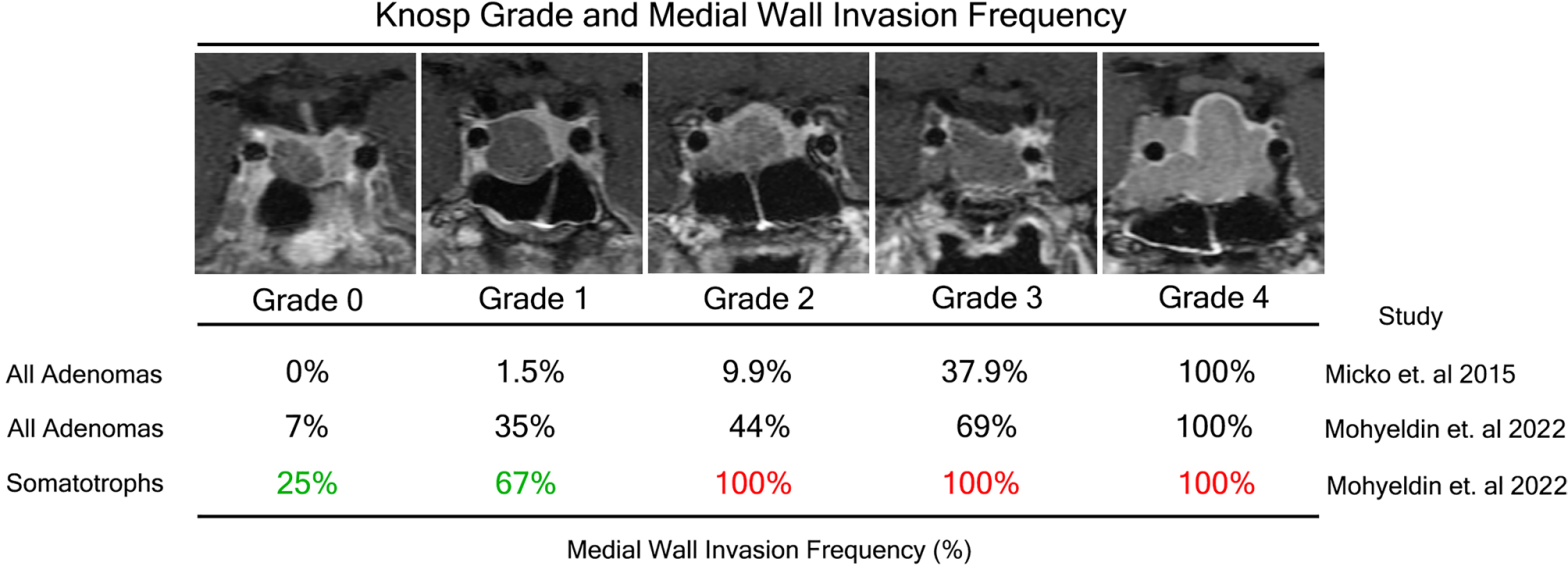
Knosp grade and medial wall invasion frequency as reported by Knosp and colleagues compared to invasion frequencies exhibited in the current study among all adenomas and then among somatotroph adenomas. Representative MRI images from case examples of somatotrophs in our current series stratified by Knosp grade. Knosp criteria were applied based on carotid tangents and each grade is represented by a coronal T1 MRI scan with gadolinium capturing the extent of cavernous sinus invasion.

**Figure 4:**
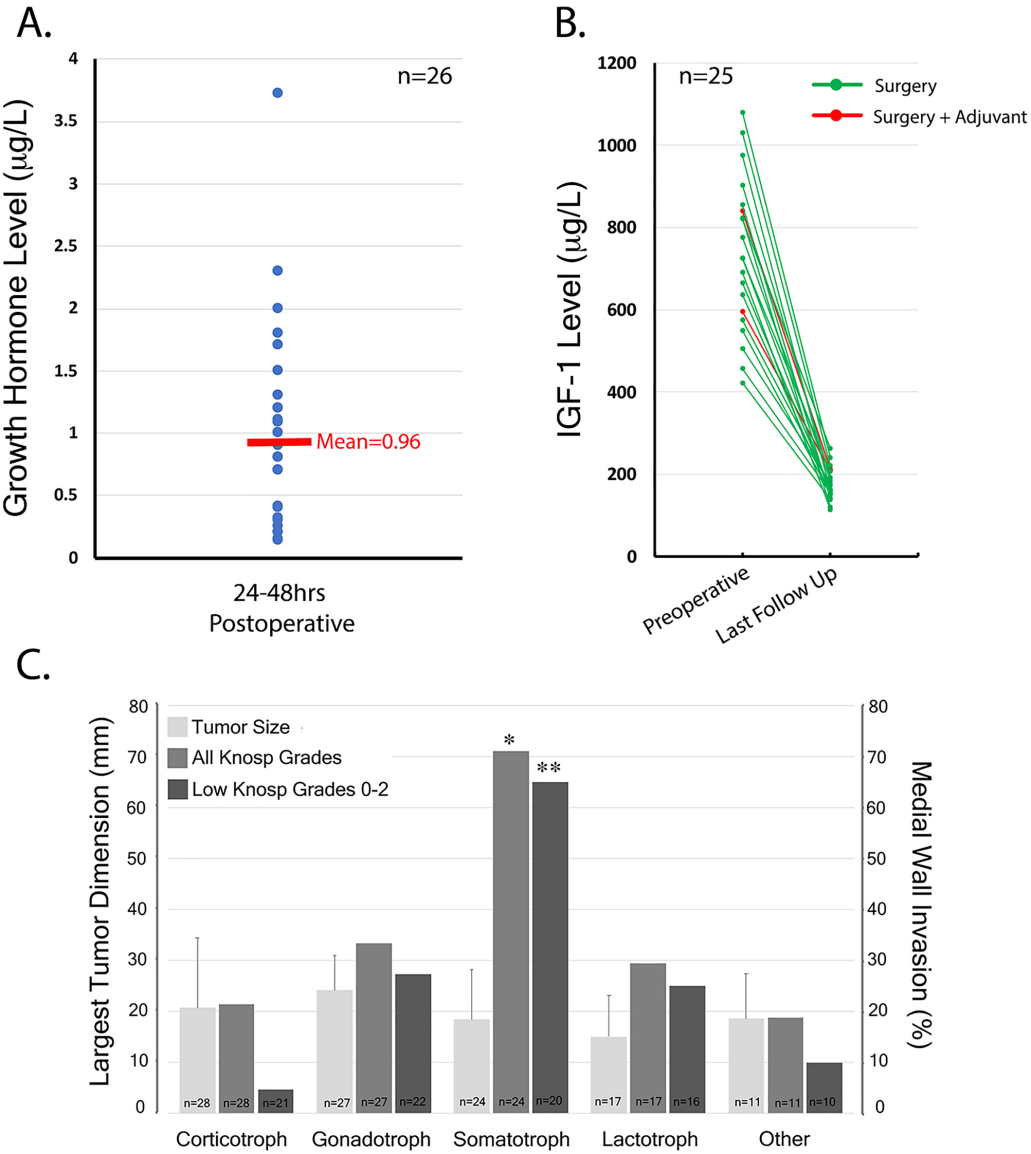
(**A**) Mean postoperative GH nadir 24-48hrs after surgical resection in acromegaly cohort (n=26) (**B**) Preoperative and postoperative (3-12 months) IGF-1 levels in acromegaly cohort (n=25), two-tailed unpaired t-test demonstrates statistically significance between preoperative and postoperative IGF-1 levels (*p*<0.0001) (**C**) A graphical visual summary of the important findings from Table 1, 2 and 3 and demonstrates that somatotroph adenomas have a predilection to invade the cavernous sinus irrespective of tumor size. Using a Chi square test, we demonstrate that the effect that somatotrophs have on predicting cavernous sinus invasion was present across all Knosp grades 0-4 (*p<0.02) and even present across low Knosp grades 0-2 (**p<.05).

When somatotroph adenomas (n=24) were stratified based on Knosp grade, histological medial wall invasion frequencies were: Knosp Grade 0=25%, 1=67%, 2=100%, 3=100%, 4=100% (Fig. 3). Compared to corticotroph adenomas which displayed a lower propensity for cavernous sinus invasion, Knosp grades 0-1 somatotroph adenomas were more likely to invade the cavernous sinus, 65% versus 4.7% (*p*<0.05). These invasion frequencies were particularly higher than the invasion frequencies observed across the entire cohort and uniquely elevated for lower Knosp grades. Figure 3 and 4C are a visual and graphical summary of the findings from Tables 1, 2 and 3 demonstrating that somatotroph adenomas have a predilection to invade the cavernous sinus irrespective of tumor size. This effect was not seen with any other pituitary adenoma subtype. Using a Chi square test, we demonstrate that the effect that somatotrophs have on predicting cavernous sinus invasion was present across all Knosp grades 0-4 (*p<0.02) and even present across low Knosp grades 0-2 (**p<.05), a finding that is not predicted by the Knosp classification itself. This effect was not seen with any other pituitary adenoma subtype.

### Acromegaly patient cohort and tumor characteristics

Given the discovery that somatotrophs have a propensity for cavernous sinus dural invasion, we further explored the effects of medial wall resection on biochemical remission in acromegaly. We retrospectively reviewed patient charts with biochemically confirmed acromegaly. Consecutive patients with newly diagnosed acromegaly (n=26) were treated with endoscopic transsphenoidal surgery between July 2018 and December 2020. Among this group 11 (42%) were female and 15 (58%) were male (Table 4), and the mean age for this cohort was 50 ± 16.3 years. The average tumor size was 18.42 ± 9.82 mm. Preoperative IGF-1 levels for this cohort were 701.5 ± 201.9 mg/L (Table 4). Four patients (15%) fulfilled the criteria for Grade 3 or 4 while 22 patients (85%) fulfilled the criteria for Grade 0-2. Based on the 2017 WHO classification, sparsely granulated adenoma subtype had the largest representation in this cohort with 10 patients (38%) followed by mixed somatolactotroph (7 patients, 27%), densely granulated (4 patients, 15%), plurihormonal adenoma (3 patients, 12%) and mammosomatotroph adenoma (2 patients, 7%). There were 21 out of 26 (81%) patients who had intraoperative evidence of medial wall invasion, which was resected. Histological analysis of resected medial walls identified 18 out of 26 (69%) tumors had pathological evidence of invasion, with a positive predictive value of 85% (18/21).

**Table 4:** Acromegaly Patient Cohort n=26.

Age (years)	Mean	SD
	49.3	15.5
Tumor size (mm)	Mean	SD
	17.8	9.7
Gender	N	Percent
F	11	42%
M	15	58%
Medial walls resected	N	Percent
	21/26	81%
IGF-1 levels	Mean	SD
Preoperative	701.5	201.9
Postoperative	194.6	54.7
Growth hormone levels	Mean	SD
Postoperative	0.96	0.8
WHO 2017 adenoma classification	N	Percent
Densely granulated	4	16%
Sparsely granulated	10	42%
Mixed somatolactotroph	7	23%
Mammosomatotroph	2	6%
Plurihormonal	3	12%
Knosp grade	N	Percent
Grade 0	6	23%
Grade 1	12	46%
Grade 2	4	15%
Grade 3A	0	0%
Grade 3B	2	8%
Grade 4	2	8%
Remission rates	N	Percent
Biochemical remission (Surgery)	23/25	92%
Biochemical remission (Surgery + Adjuvant)	2/25	8%
Biochemical remission of entire case series	25/25	100%

Cohort of 26 consecutive newly diagnosed acromegaly patients. Variables include age, tumor size, gender, initial presentation, number of medial walls resected, postoperative growth hormone levels, preoperative and postoperative IGF-1 levels, WHO 2017 Adenoma subtype classification, Knosp grade and remission rates. Categorical variables are reported as number and percentage, and continuous variables are reported as mean ± standard deviation.

### Postoperative surgical and endocrinological outcomes in acromegaly

No patients who underwent endoscopic transsphenoidal surgery in the acromegaly cohort were taking somatostatin analogs, GH receptor antagonists or dopamine agonists at or around the time of their surgery and no patients received radiation therapy prior to surgery. All 26 patients had post-operative GH levels measured at 24-48 hrs after surgery and 25/26 patients had follow-up GH levels (≥6 months) with IGF-1 levels measured at 6 weeks, 3 months and/or 6 months post op. The mean post-operative GH levels measured at 24-48 hr for this cohort was 0.96 ± 0.8 mg/L (Fig. 4A). The mean post-operative IGF-1 levels measured at 3-12 months for this cohort was 194.6 ± 54.7 mg/L; when compared to preoperative IGF-1 levels (701.5 ± 201.9 mg/L) using a two-tailed unpaired t-test this was statistically significant (*p*<0.0001, Fig. 4B). Remission was achieved in 23/25 (92%) patients with surgery alone (Fig. 4B). Two of 25 patients who were not in biochemical remission at 6 months after surgery required adjuvant therapy (1 patient received radiation and medication, 1 patient required medication only, Fig. 4B). Disease control was achieved in 25/25 patients (100%) either with surgery alone (92%) or surgery with adjuvant therapy (8%) (Fig. 4B). There was only one patient that was lost to follow up in this cohort. Further sub-analysis for post-operative GH levels and change in IGF-1 levels was performed to look for any potential differences in this cohort, particularly between tumors that were deemed to be invasive intraoperatively vs. non-invasive intraoperatively. We did not find any statistical significance between these two groups for GH levels or change in IGF-1 levels respectively (*p*=0.47036 and *p*=0.39400).

### Postoperative complications

The safety of medial wall resection and cavernous sinus surgery has been well documented in previous reporting from our group^13^. In 107 primary pituitary adenomas with 50 resected medial walls, we did not experience any internal carotid injury, postoperative hematoma, or pseudoaneurysm. Four patients experienced post-operative transient diplopia as a neurological deficit involving the abducens nerve, two of whom recovered within one week, while the other two recovered by one month follow-up. All four (3.7%) patients with diplopia had Knosp Grade 3 or 4 tumors preoperatively. Two patients (1.9%) had a post-operative CSF leak which required primary surgical repair with a nasal septal flap. No patients developed new panhypopituitarism. Rates of diabetes insipidus (DI), syndrome of inappropriate anti-diuretic hormone (SIADH) and single hormone endocrinopathies are not reported as they are beyond the aim of this study.

## Discussion

In this prospective study, we use intraoperative visualization of the medial wall of the cavernous sinus followed by direct histopathological evaluation of resected medial wall specimens and discovered that different pituitary adenoma subtypes have a varying predilection to invade the cavernous sinus. Somatotroph adenomas exhibit the highest frequency of invasion at all Knosp grades examined. These findings affirm previous observations^5,13,21,22,31^, however raise questions about some long-held assumptions about the frequency of cavernous sinus invasion among pituitary adenoma subtypes.

Previous studies that have aimed to identify such patterns of invasion largely relied on retrospective analyses which have inherent biases or utilize nonconsecutive specimen collection^20,21,23^. Our data was collected in a prospective manner and while some investigators have prospectively collected dural specimens they did not examine the medial wall of the cavernous sinus histologically^4^. Sampling sellar floor dura or anterior dura from the face of the sella is not an accurate surrogate for cavernous sinus invasion, making it difficult to draw direct conclusions from such data. Histopathological examination of the medial wall itself has previously been reported to be the gold standard for evaluating cavernous sinus invasion underscoring the interpretations of our findings and their implications. The high reliability of intraoperative observation in predicting medial wall invasion is yet another testament to the benefit of using endoscopes, in particular angled endoscopes for direct observation laterally to each side, over the historic microscopic view when performing pituitary surgery. Given how reliable intraoperative observation is with high resolution endoscopes, false negatives in our cohort were a result of coagulation artifact of the medial wall during resection and possible sampling error in cases when *en bloc* resection was not feasible. The positive predictive value in our case series (78%) is higher than that reported by other groups and may certainly be improved up on in future studies by reducing coagulation artifact during medial wall resection as surgeons become more proficient in this technique. In addition, the potential for intraoperative Raman spectroscopy in sampling the medial wall for invasion during pituitary surgery may offer live feedback to the decision-making process intraoperatively, but the methodologies behind such technology need to be validated and worked out^32^.

The extent of cavernous sinus invasion by pituitary adenomas remains a significant predictor of incomplete surgical resection rates, lack of biochemical remission and tumor recurrence^4–6,9,14,15^ The Knosp classification aims to predict the probability of cavernous sinus involvement based on increasing extension of adenomas past predetermined tangent lines of the carotid artery on MRI examination^5,16^. In their case series of consecutive pituitary adenomas evaluating medial wall invasion intraoperatively using an endoscope, Knosp and colleagues^5^ discovered the following invasion rates for each Knosp grade: 0=0%, 1=1.5%, 2=9.9%, 3a=26.5%, 3b=70.6% and 4=100%. It is important to note that they did not perform surgical resection of invaded medial walls but rather confined their resection only to gross tumor invading the cavernous sinus when it was visualized intraoperatively; this limited their ability to evaluate microscopic invasion histologically as we performed in our study.

Both intraoperative evaluation of the medial wall and histopathological analysis from our study affirm the same escalating trend in cavernous sinus invasion rate with increasing Knosp grade but with higher invasion rates than reported by Knosp and colleagues (Fig. 3). While the Knosp study^5^ did not evaluate the effect that pituitary adenoma subtype had on predicting invasion, in our multivariate analysis we discovered that somatotroph adenomas predicted cavernous sinus invasion (intraoperatively *p*<0.02, histologically *p*<0.05). In fact, the frequency of invasion for somatotroph adenomas based on both techniques in our cohort was significantly higher when compared to the entire cohort for both high and low Knosp grades (Fig. 4C).

There are notable differences between our study and the Knosp study that may explain such diversity in invasion frequencies. The Knosp cohort^5^ only had 10% representation of somatotrophs while in our study, somatotrophs made up 22% of the entire cohort. If somatotrophs indeed have a predilection for cavernous sinus invasion compared to other adenoma subtypes as evidenced by our histopathological analysis, this would result in lower invasion frequencies in a cohort where they are relatively under-represented. Another variable that can account for such findings between the two studies is the overwhelming representation of null cell adenomas in the Knosp cohort (45%) compared to our study (5%). Null cell adenomas showed a low propensity to invade the cavernous sinus in our cohort which can reduce invasion frequencies across all Knosp grades in a study where they are over-represented. Our findings underscore that the Knosp grading system significantly underpredicts the frequency of invasion in somatotroph adenomas, particularly at low Knosp grades (Knosp 0-2). The indiscriminate use of the Knosp classification system across all pituitary adenoma subtypes needs to be reexamined as our data points to significant diversity among invasion frequencies. Surgeons should have a higher suspicion for cavernous sinus invasion among somatotroph adenomas and these findings for the first time may explain the mechanism behind the failed biochemical remission rates seen in acromegaly over other functional tumors.

The remission rate in acromegaly after surgery varies and is currently reported to range from 32 to 85%^15,33^. A meta-analysis published in 2016 using the most current consensus statements indicated pooled remission rates of 55%^33^. The highest biochemical remission rates reported in the literature have come from Yamada and colleagues who treated 150 consecutive acromegaly patients and found that 55 (36.7%) patients had intraoperative evidence of cavernous sinus involvement^22^. This cavernous sinus invasion rate in acromegaly is nearly double the invasion frequency identified by Knosp and colleagues across all adenomas in their study (16.6%). This underscores our timely observation that somatotrophs invade the cavernous sinus more than other adenoma subtypes. In our study, we identified a higher invasion rate among acromegaly patients than that reported by Yamada with 69% and 80.7% invasion frequencies confirmed via histological and intraoperative evaluation respectively. We suspect that improved visualization with higher resolution endoscopes, advanced surgical techniques in the exposure and resection of the medial wall of the cavernous sinus and direct histological evaluation of the medial contributed to the higher observation of invasion seen in our acromegaly cohort. It is important to note that when Yamada’s group was able to send the medial wall for histological evaluation, invasion was confirmed in 16 of the 18 specimens sent (89%). These findings collectively set an important precedent for careful evaluation of the medial wall of the cavernous sinus in the surgical treatment of acromegaly and particularly with low Knosp grades.

Our biochemical remission rates with surgery alone (92%) and surgery with adjuvant therapy (100%) are significantly higher than previously reported rates but longer follow-up and a larger cohort are required for definitive conclusions. A previous study that looked at post-operative GH nadirs as an index of long-term remission showed that a GH nadir lower than 1.15 ug/L provided the best predictor of remission with a sensitivity of 73%, specificity of 85% and a positive predictive value of 54%^14^. Our mean 24-48hr GH nadir was 0.96 ± 0.78 mg/L for our acromegaly cohort, which is highly suggestive of durable biochemical remission. Although achieving biochemical remission with surgery maybe challenging in acromegaly^33^, long-term follow up of acromegaly patients suggests recurrence is rare once remission is achieved when compared to prolactinomas and Cushing’s disease adenomas^34^. Nomikos et al. identified 2 recurrent cases (0.4%) in their series of 668 acromegaly patients during a mean follow-up period of more than 10 years^35^.

There still remains considerable controversy about removal of the medial wall and resection of cavernous sinus disease in other invasive pituitary adenoma subtypes. There are several observations that make a reappraisal of this position compelling. Only 75% of prolactinomas that exhibit Knosp grades 3 or 4 can be controlled biochemically with multimodality treatment^36^. A meta-analysis looking at the use of stereotactic radiosurgery (SRS) and fractionated stereotactic radiosurgery (FSRS) in the management of nonfunctioning pituitary adenomas has shown promising tumor control rates with 94% and 83% control rates at 5 and 10 years out^37^. Unfortunately, related radio-toxicities with respect to new hypopituitarism, visual decline and associated cranial neuropathies were noted and these are not trivial side effects^37^. Furthermore, emerging evidence from the newly recognized WHO 2017 criteria suggests that silent corticotrophs and plurihormonal adenomas may represent a subset of nonfunctioning adenomas that maybe refractory to SRS^38,39^.

In conclusion, adenomas that invade the medial wall of the cavernous sinus should be evaluated on a case-by-case basis and removed by experienced surgeons particularly when endocrinological remission is at stake. Our findings corroborate previously published reports with regards to safety and outcomes. These results offer the potential to improve surgical outcomes in neuroendocrine tumors; particularly in acromegaly, where somatotroph adenomas now appear to have an intrinsic propensity to invade the cavernous sinus wall with very high frequency across both high and low Knosp grades. The increasing use of stereotactic radiation to treat cavernous sinus disease has had mixed results with a significant time interval to achieve remission from treatment onset^40–46^. Furthermore, the morbidity associated with permanent cranial neuropathies with radiation may not be better than the safety profile of medial wall resection by experienced groups, although more research is warranted in this area^40^. Finally, both SRS and cavernous sinus surgery should be perceived as complementary techniques with synergistic efficacy rather than competing treatment paradigms.

## References

[1] Lopes MBS (2017). The 2017 world health organization classification of tumors of the pituitary gland: a summary. Acta. Neuropathol..

[2] Melmed S (2020). Pituitary-tumor endocrinopathies. N. Engl. J. Med..

[3] WHO classification of tumours of endocrine organs, 4th edn. Lyon, France: IARC Press 2017.

[4] Lonser RR, Ksendzovsky A, Wind JJ, Vortmeyer AO, Oldfield EH (2012). Prospective evaluation of the characteristics and incidence of adenoma-associated dural invasion in Cushing disease. J. Neurosurg..

[5] Micko AS, Wohrer A, Wolfsberger S, Knosp E (2015). Invasion of the cavernous sinus space in pituitary adenomas: endoscopic verification and its correlation with an MRI-based classification. J. Neurosurg..

[6] Micko A, Oberndorfer J, Weninger WJ (2019). Challenging Knosp high-grade pituitary adenomas. J. Neurosurg..

[7] Scheithauer BW, Kovacs KT, Laws ER, Randall RV (1986). Pathology of invasive pituitary tumors with special reference to functional classification. J. Neurosurg..

[8] Chatzellis E, Alexandraki KI, Androulakis II, Kaltsas G (2015). Aggressive pituitary tumors. Neuroendocrinology.

[9] Zada G, Lin N, Laws ER (2010). Patterns of extrasellar extension in growth hormone-secreting and nonfunctional pituitary macroadenomas. Neurosurg. Focus.

[10] Neou M, Villa C, Armignacco R (2020). Pangenomic classification of pituitary neuroendocrine tumors. Cancer Cell.

[11] Yasuda A, Campero A, Martins C, Rhoton AL, Ribas GC (2004). The medial wall of the cavernous sinus: Microsurgical anatomy. Neurosurgery.

[12] Truong HQ, Lieber S, Najera E, Alves-Belo JT, Gardner PA, Fernandez-Miranda JC (2018). The medial wall of the cavernous sinus. Part 1: Surgical anatomy, ligaments, and surgical technique for its mobilization and/or resection. J. Neurosurg..

[13] Cohen-Cohen S, Gardner PA, Alves-Belo JT (2018). The medial wall of the cavernous sinus. Part 2: Selective medial wall resection in 50 pituitary adenoma patients. J. Neurosurg..

[14] Starke RM, Raper DM, Payne SC, Vance ML, Oldfield EH, Jane JA (2013). Endoscopic vs microsurgical transsphenoidal surgery for acromegaly: outcomes in a concurrent series of patients using modern criteria for remission. J. Clin. Endocrinol. Metab..

[15] Agrawal N, Ioachimescu AG (2020). Prognostic factors of biochemical remission after transsphenoidal surgery for acromegaly: A structured review. Pituitary.

[16] Knosp E, Steiner E, Kitz K, Matula C (1993). Pituitary adenomas with invasion of the cavernous sinus space: a magnetic resonance imaging classification compared with surgical findings. Neurosurgery.

[17] Selman WR, Laws ER, Scheithauer BW, Carpenter SM (1986). The occurrence of dural invasion in pituitary adenomas. J. Neurosurg..

[18] Thapar K, Kovacs K, Scheithauer BW (1996). Proliferative activity and invasiveness among pituitary adenomas and carcinomas: An analysis using the MIB-1 antibody. Neurosurgery.

[19] Oldfield EH (2017). Cushing's disease: Lessons learned from 1500 cases. Neurosurgery.

[20] Dickerman RD, Oldfield EH (2002). Basis of persistent and recurrent Cushing disease: an analysis of findings at repeated pituitary surgery. J. Neurosurg..

[21] Nishioka H, Fukuhara N, Horiguchi K, Yamada S (2014). Aggressive transsphenoidal resection of tumors invading the cavernous sinus in patients with acromegaly: predictive factors, strategies, and outcomes. J. Neurosurg..

[22] Nagata Y, Takeuchi K, Yamamoto T (2019). Removal of the medial wall of the cavernous sinus for functional pituitary adenomas: A technical report and pathologic significance. World Neurosurg..

[23] Meij BP, Lopes MB, Ellegala DB, Alden TD, Laws ER (2002). The long-term significance of microscopic dural invasion in 354 patients with pituitary adenomas treated with transsphenoidal surgery. J. Neurosurg..

[24] Mooney MA, Hardesty DA, Sheehy JP (2017). Rater reliability of the hardy classification for pituitary adenomas in the magnetic resonance imaging era. J. Neurol. Surg. B Skull Base.

[25] Micko A, Oberndorfer J, Weninger WJ (2019). Challenging Knosp high-grade pituitary adenomas. J. Neurosurg..

[26] Giustina A, Chanson P, Bronstein MD (2010). A consensus on criteria for cure of acromegaly. J. Clin. Endocrinol. Metab..

[27] Cai X, Zhu J, Yang J (2021). A nomogram for preoperatively predicting the Ki-67 index of a pituitary tumor: A retrospective cohort study. Front. Oncol..

[28] Paek KI, Kim SH, Song SH (2005). Clinical significance of Ki-67 labeling index in pituitary macroadenoma. J. Korean Med. Sci..

[29] Grimm F, Maurus R, Beschorner R (2019). Ki-67 labeling index and expression of p53 are non-predictive for invasiveness and tumor size in functional and nonfunctional pituitary adenomas. Acta Neurochir. (Wien).

[30] Yuhan, L., Zhiqun, W., Jihui, T., Renlong, P. Ki-67 labeling index and Knosp classification of pituitary adenomas. Br. J. Neurosurg.1-5 (2021). 10.1080/02688697.2021.1884186 33905276

[31] Almeida JP, Ruiz-Trevino AS, Liang B (2018). Reoperation for growth hormone-secreting pituitary adenomas: report on an endonasal endoscopic series with a systematic review and meta-analysis of the literature. J. Neurosurg..

[32] Hollon TC, Pandian B, Adapa AR (2020). Near real-time intraoperative brain tumor diagnosis using stimulated Raman histology and deep neural networks. Nat. Med..

[33] Starnoni D, Daniel RT, Marino L, Pitteloud N, Levivier M, Messerer M (2016). Surgical treatment of acromegaly according to the 2010 remission criteria: systematic review and meta-analysis. Acta Neurochir. (Wien).

[34] Nishioka H, Yamada S (2014). Response. J. Neurosurg..

[35] Nomikos P, Buchfelder M, Fahlbusch R (2005). The outcome of surgery in 668 patients with acromegaly using current criteria of biochemical 'cure'. Eur. J. Endocrinol..

[36] Wu ZB, Su ZP, Wu JS, Zheng WM, Zhuge QC, Zhong M (2008). Five years follow-up of invasive prolactinomas with special reference to the control of cavernous sinus invasion. Pituitary.

[37] Kotecha R, Sahgal A, Rubens M (2020). Stereotactic radiosurgery for non-functioning pituitary adenomas: meta-analysis and international stereotactic radiosurgery society practice opinion. Neuro. Oncol..

[38] Cohen-Inbar O, Xu Z, Lee CC (2017). Prognostic significance of corticotroph staining in radiosurgery for non-functioning pituitary adenomas: a multicenter study. J. Neurooncol..

[39] Xu Z, Ellis S, Lee CC (2014). Silent corticotroph adenomas after stereotactic radiosurgery: a case-control study. Int. J. Radiat. Oncol. Biol. Phys..

[40] Ding D, Mehta GU, Patibandla MR (2019). Stereotactic radiosurgery for acromegaly: An international multicenter retrospective cohort study. Neurosurgery.

[41] Shrivastava A, Mohammed N, Xu Z (2019). Outcomes after gamma knife stereotactic radiosurgery in pediatric patients with cushing disease or acromegaly: A multi-institutional study. World Neurosurg..

[42] Franzin A, Spatola G, Losa M, Picozzi P, Mortini P (2012). Results of gamma knife radiosurgery in acromegaly. Int. J. Endocrinol..

[43] Liu X, Kano H, Kondziolka D (2012). Gamma knife radiosurgery for clinically persistent acromegaly. J. Neurooncol..

[44] Mohammed N, Ding D, Hung YC (2019). Primary versus postoperative stereotactic radiosurgery for acromegaly: A multicenter matched cohort study. J. Neurosurg..

[45] Losa M, Gioia L, Picozzi P (2008). The role of stereotactic radiotherapy in patients with growth hormone-secreting pituitary adenoma. J. Clin. Endocrinol. Metab..

[46] Castinetti F, Taieb D, Kuhn JM (2005). Outcome of gamma knife radiosurgery in 82 patients with acromegaly: Correlation with initial hypersecretion. J. Clin. Endocrinol. Metab..

